# Effects of MS-153 on chronic ethanol consumption and GLT1 modulation of glutamate levels in male alcohol-preferring rats

**DOI:** 10.3389/fnbeh.2014.00366

**Published:** 2014-10-30

**Authors:** Hasan Alhaddad, Nathaniel T. Kim, Munaf Aal-Aaboda, Yusuf S. Althobaiti, James Leighton, Sai H. S. Boddu, Yangjie Wei, Youssef Sari

**Affiliations:** ^1^Department of Pharmacology, College of Pharmacy and Pharmaceutical Sciences, University of ToledoToledo, OH, USA; ^2^Department of Chemistry, Columbia UniversityNew York, NY, USA; ^3^Department of Pharmacy Practice, College of Pharmacy and Pharmaceutical Sciences, University of ToledoToledo, OH, USA

**Keywords:** MS-153, glutamate, EAAT2, GLT1, alcohol dependence, P rats

## Abstract

We have recently shown that upregulation of glutamate transporter 1 (GLT1) in the brain is associated in part with reduction in ethanol intake in alcohol-preferring (P) male rats. In this study, we investigated the effects of a synthetic compound, (R)-(−)-5-methyl-1-nicotinoyl-2-pyrazoline (MS-153), known to activate GLT1 on ethanol consumption as well as GLT1 expression and certain signaling pathways in P rats. P rats were given 24-h concurrent access to 15 and 30% ethanol, water and food for 5 weeks. On week 6, P rats received MS-153 at a dose of 50 mg/kg (i.p.) or a vehicle (i.p.) for 5 consecutive days. We also tested the effect of MS-153 on daily sucrose (10%) intake. Our studies revealed a significant decrease in ethanol intake at the dose of 50 mg/kg MS-153 from Day 1 through 14. In addition, MS-153 at dose of 50 mg/kg did not induce any significant effect on sucrose intake. Importantly, we found that MS-153 upregulated the GLT1 level in the nucleus accumbens (NAc) but not in the prefrontal cortex (PFC). In accordance, we found upregulation of nuclear NFkB-65 level in NAc in MS-153-treated group, however, IkBα was downregulated in MS-153-treated group in NAc. We did not find any changes in NFkB-65 and IkBα levels in PFC. Interestingly, we revealed that p-Akt was downregulated in ethanol vehicle treated groups in the NAc; this downregulation was reversed by MS-153 treatment. We did not observe any significant differences in glutamate aspartate transporter (GLAST) expression among all groups. These findings reveal MS-153 as a GLT1 modulator that may have potential as a therapeutic drug for the treatment of alcohol dependence.

## Introduction

Deficits in glutamate uptake have been suggested to impair neurocircuits involved in drug abuse and drug-seeking behavior, affecting many aspects of neuroplasticity associated with ethanol and drug addiction. A key source of dependency is thought to be alcohol-induced changes in glutamate transmission (Smith, 1997; Smith and Weiss, 1999). Ethanol-seeking behavior is promoted by increased glutamate transmission in key regions of the mesocorticolimbic reward circuit (Selim and Bradberry, 1996; Quertemont et al., 1998; Dahchour et al., 2000; Roberto et al., 2004; Melendez et al., 2005; Kapasova and Szumlinski, 2008). Moreover, *in vitro* and *in vivo* studies have demonstrated that ethanol exposure affects glutamate transport (Smith, 1997; Smith and Weiss, 1999; Othman et al., 2002; Melendez et al., 2005). It is noteworthy that glutamate transporter 1 (GLT1) is responsible for the removal of most of the extracellular glutamate (Ginsberg et al., 1995; Rothstein, 1995; Danbolt, 2001; Mitani and Tanaka, 2003). Studies have shown that ceftriaxone, a β-lactam antibiotic, has an upregulatory effects in GLT1 levels (Rothstein et al., 2005; Miller et al., 2008; Sari et al., 2009, 2010). Ceftriaxone was found to normalize the glutamate transport capacity and basal glutamate levels after chronic drug addiction (Trantham-Davidson et al., 2012). Studies from our lab and others have demonstrated that ceftriaxone attenuated cue-induced reinstatement of cocaine-seeking behavior (Sari et al., 2009; Knackstedt et al., 2010). We have recently shown that ceftriaxone treatment reduced ethanol intake in alcohol-preferring (P) rats (Sari et al., 2011, 2013a) and attenuated relapse-like to ethanol-drinking behavior (Qrunfleh et al., 2013). The behavioral effects of ceftriaxone were associated with upregulation of GLT1 in the prefrontal cortex (PFC) and nucleus accumbens (NAc).

We focused here on testing a new synthetic compound, a non-β-lactam antibiotic, which regulates glutamate transmission for potential attenuation of ethanol intake in male P rats. We also focused on the effects of this compound in GLT1 expression. Thus, we studied MS-153 [(R)-(−)-5-methyl-1-nicotinoyl-2-pyrazoline], a novel pyrazoline compound found to be a neuroprotective agent, which decreased the extracellular glutamate level in the ischemic penumbra zone during permanent occlusion of the middle cerebral artery (Kawazura et al., 1997). The compound has been suggested to act as a GLT1 activator, which increases the activity of GLT1 and thus accelerates glutamate uptake (Shimada et al., 1999). MS-153 has the ability to enhance glutamate uptake or decrease glutamate release, which both attenuate the development of behavioral sensitization to phencyclidine-induced stereotypies (Abekawa et al., 2002). Studies have demonstrated that administration of MS-153 decreased the development of morphine tolerance and physical dependence in mouse models (Nakagawa et al., 2001). Furthermore, administration of MS-153 attenuated the induction of conditioned place preference to morphine, methamphetamine and cocaine without affecting acute locomotor responses (Nakagawa et al., 2001, 2005). In this study, we tested whether MS-153 treatment would attenuate ethanol intake in P rats. We also determined the effect of MS-153 on sucrose intake as an appetitive control for drinking-motivated behavior in male P rats. We further determined the effects of MS-153 on GLT1 levels in both PFC and NAc. We then focused our study on investigating certain signaling pathways involving MS-153 in the regulation of GLT1 levels in the NAc and PFC. Based on recent studies showing the existence of a link between GLT1 and NF-kB and Akt pathways (Lee et al., 2008; Wu et al., 2010), we endeavored to investigate the effects of MS-153 on these pathways. We also determined the effects of MS-153 in glutamate aspartate transporter (GLAST) levels in PFC and NAc.

## Materials and methods

### MS-153 synthesis

**Figure 1:**
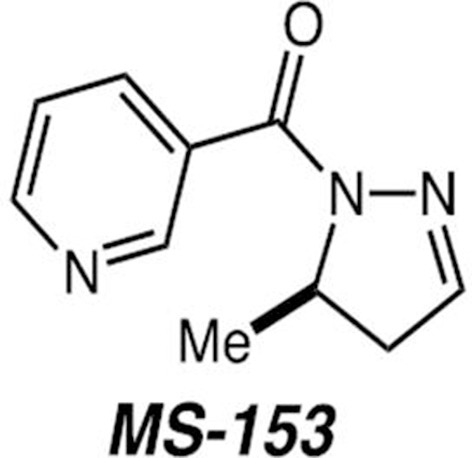
**(R)-(−)-5-methyl-1-nicotinoyl-2-pyrazoline (MS-153) structure**.

MS-153 [(R)-(−)-5-methyl-1-nicotinoyl-2-pyrazoline] (Figures 1) was synthesized in-house at Columbia University using a chiral silane Lewis acid-promoted acylhydrazone-enol ether [3 + 2] cycloaddition that delivers the pyrazolidine product in 98% enantiomeric excess (ee). Nicotinoylation of the pyrazolidine product is followed by cleavage of the *p*-nitrobenzamide and concomitant *t*-butoxide elimination to provide MS-153 in 70% overall yield for the three-step sequence. Full experimental details regarding MS-153 synthesis can be found in the previous study by our group (Tran and Leighton, 2006).

### Analytical procedure and quantitative determination of M-153 in male P rat plasma and CSF

A high-performance liquid chromatography system (HPLC) (Waters Alliance 2695 separation module, Milford, MA) equipped with a Kinetex C18 column (250 × 4.6 mm, Phenomenex) and UV/Visible detector was used for analysis. MS-153 was analyzed by an isocratic method with a mobile phase containing water and acetonitrile (85:15) pumped at a flow rate of 1 ml/min. The retention time of MS-153 (λ_*max*_ = 260 nm) was found to be 7 min. Different calibration standards of MS-153 were prepared in the mobile phase. For the calibration curve, each standard was analyzed in triplicate, and the average peak area was plotted against concentration. The drug content was determined quantitatively by plotting a calibration curve. The assay method was found to be linear in the range of 0.015625–10 μg/ml, with a correlation coefficient of 0.9999. The percentage recovery of MS-153 ranged from 99.97 to 101.66%. The limit of detection and limit of quantification of MS-153 were found to be 2.98 and 9.92 ng/ml, respectively. The intra- and inter-day precisions (measured by %RSD) were found to be in the ranges of 0.21–0.55% and 0.32–0.82%, respectively. Plasma and CSF samples were mixed with equal amounts of methanol and vortexed for 30 s. The mixture was then centrifuged at 5000 rpm for 10 min. The supernatant was further analyzed using HPLC.

### Animals and ethanol drinking paradigm

Male adult P rats were received from Indiana University School of Medicine, Indianapolis, at the age of 21–30 days. Rats were housed in the Department of Laboratory Animal Resources, University of Toledo, in standard plastic tubs with corn-cob bedding and had ad lib access to chow food and water throughout the experimental procedures. P rats were acclimated at a temperature of 25°C and 50% humidity in a 12-h light/dark cycle. We used P rats as a model of ethanol dependence, which demonstrated in previous studies (Stewart et al., 1991). Animal experimental procedures were approved by the Institutional Animal Care and Use Committee of The University of Toledo in accordance with the guidelines of the Institutional Animal Care and Use Committee of the National Institutes of Health and the Guide for the Care and Use of Laboratory Animals (Institute of Laboratory Animal Resources, Commission on Life Sciences, 1996). The programs and procedures are accredited by the Association for the Assessment and Accreditation of Laboratory Animal Care, International.

P rats started the experimental procedures at the age of 90 days old. Three experimental groups were tested for the effects of MS-153 on ethanol intake, water intake and body weight. (1) The naïve ethanol vehicle (Naïve) group was exposed to water and food only and received i.p. injections of vehicle solution (1% DMSO in PBS), *n* = 11; (2) Ethanol vehicle group (ethanol control group) received i.p. injections of vehicle, *n* = 15; and (3) Ethanol MS-153 group received a dose of 50 mg/kg, i.p., *n* = 13. Ethanol vehicle and ethanol MS-153 groups had free-choice access to water, 15 and 30% ethanol and food throughout the experimental procedures.

At the age of 90 days, male P rats were exposed to continuous access of free choice of ethanol (15 and 30%, v/v, concurrently), water and *ad libitum* food for 5 weeks. The naïve ethanol vehicle group had access to *ad libitum* food and water. For the ethanol vehicle and ethanol MS-153 groups, ethanol consumption was measured as grams of ethanol consumed per kilogram of rat body weight per day for 5 weeks. The average measurements taken across the last 2 weeks of the 5 weeks of consumed ethanol and water were calculated as the baseline. The average measurements of ethanol intake were based on cumulative consumption of 15 and 30% ethanol. The measurements of consumed ethanol were evaluated to the nearest ^1^/_10_ of a gram by subtracting the bottle weight from its initial weight containing ethanol. These measurements were converted using a densitometry formula to the actual grams of consumed ethanol per kg of rat body weight per day. On week six, rats were injected with 50 mg/kg of MS-153 or with vehicle at around 11:00 a.m. once a day for 5 days. Ethanol intake, water intake and rat body weight were measured daily for 14 days, starting on the first day of the vehicle and MS-153 i.p. injections.

### Sucrose drinking paradigm

We further tested the effects of MS-153 on sucrose (10%) intake as an appetitive control, drinking-motivated behavior. Animals were exposed to continuous access to 10% sucrose solution, water, and food for 3 weeks. On Week 4, a group of P rats (*n* = 7) received the vehicle and another group (*n* = 5) received 50 mg/kg of MS-153 (i.p.) for 5 consecutive days. Sucrose was available 6 days after the first day of MS-153 and vehicle i.p. injections. Body weight and sucrose and water intake were measured daily for 6 days starting on the first day of the vehicle or MS-153 i.p. injections.

### Brain tissue harvesting

On the last day of the drinking paradigm of each study, all the animals were euthanized by CO_2_ inhalation and rapidly decapitated with a guillotine; their brains were dissected and immediately frozen on dry ice and stored at −70°C. The PFC and NAc were microdissected stereotaxically using a cryostat apparatus, as described recently (Sari and Sreemantula, 2012). We followed the stereotaxic coordinates for the rat brain for identification of the PFC and NAc (Paxinos and Watson, 2007). We used surgical blades to isolate the PFC and NAc through visualized landmarks. The PFC (medial part) was isolated at the same level of NAc. The PFC and NAc were extracted for Western blot procedures for detection of GLT1, GLAST, NF-kB-p65, IkBα, phospho-Akt, Akt, Glyceraldehyde-3-phosphate Dehydrogenase (GAPDH), lamin, and β-tubulin.

### Western blot protocol for detection of GLT1, GLAST, phospho-Akt and total-Akt

GLT1, GLAST, β-tubulin, total-Akt, phospho-Akt, and Glyceraldehyde-3-phosphate Dehydrogenase (GAPDH) levels were determined in NAc and PFC of the naïve ethanol, ethanol vehicle, and ethanol MS-153 groups using Western blot assay. The NAc and PFC were homogenized in lysis buffer containing protease inhibitor and phosphatase inhibitor. Proteins from each brain region were extracted for immunoblotting. Equal amounts of extracted protein were mixed with 5x Laemmli loading dye and further separated in 10–20% glycine gel (Life Technologies). The proteins were then transferred onto nitrocellulose membranes using a transfer apparatus. Membranes were incubated overnight in blocking buffer at 4°C with one of the following antibodies: guinea pig-anti GLT1 antibody (1:5000; Millipore), rabbit anti-GLAST (dilution: 1:5000), rabbit anti-phospho-Akt (1:5000, Cell Signaling Technology), mouse anti-Akt (1:5000, Cell Signaling Technology), mouse anti β-tubulin antibody (1:5000; Covance), or mouse anti-GAPDH antibody (1:5000; Millipore). Membranes were then washed and incubated with horseradish peroxidase (HRP) donkey-anti-Guinea pig IgG (H + L) secondary antibody (1:5000), anti-mouse IgG, HRP-linked secondary antibody (1:5000), or anti-rabbit IgG, HRP-linked secondary antibody (1:5000). Membranes were incubated with a chemiluminescent kit (SuperSignal West Pico) for protein detection. Membranes were then exposed to Kodak BioMax MR films (Thermo Fisher Scientific), and films were developed using an SRX-101A machine. Blots for each detected protein were digitized and quantified using an MCID system. Data were calculated as ratios of GLT1/β-tubulin, GLAST/GAPDH, and phospho-Akt/total-Akt.

### Western blot for detection of NF-kB-p65 and IkBα in nuclear or cytoplasmic fraction

Brain samples (NAc and PFC) were homogenized in buffer A [10 mM HEPES-KOH, pH 7.9; 1.5 mM MgCl2; 10 mM KCl; 1 mM Dithiothreitol (DDT); 1 mM phenylmethylsulfonyl fluoride (PMSF); 10 uL of protease inhibitor cocktail/ml of buffer] and then incubated at 4°C for 10 min before addition of Nonidet P40 to a final concentration of 0.1%. Brain samples were then incubated for 2 min at 4°C and centrifuged at 13,200 rpm for 15 min at 4°C. The supernatant was removed, and NaF, Na-vanadate and Na-pyrophosphate were added to the final concentrations of 50, 10, and 0.1 mM, respectively, to obtain the cytosolic fraction. The pellet was re-suspended in another buffer [20 mM HEPES-KOH, pH 7.9; 25% glycerol; 420 mM NACl; 1.5 mM MgCl2; 1 mM DDT; 1 mM PMSF; 0.2 mM EDTA; 50 mM NaF; 10 mM Na vanadate; 0.1 mM Na pyrophosphate; 10 uL of protease inhibitor cocktail/ml of buffer] and incubated in ice for 30 min. The samples were then centrifuged at 13,200 rpm at 4°C, and supernatant was collected as the nuclear fraction.

Using Western blot as described earlier, we detected NF-kB, IkBα, Lamin, and GAPDH. We used the following antibodies to detect these proteins: anti-GAPDH antibody (1:5000; Millipore), Anti IkB-α (1:500; Santa Cruz biotechnology, Inc.), anti Lamin A/C (1:5000; Santa Cruz biotechnology, Inc.), and NF-kB p65 antibody (1:500; Cell signaling technology).

### Statistical analysis

General Linear Model (GLM) repeated measures were used in this study for statistical analysis (SPSS) of ethanol consumption, water and sucrose intake, and body weight measurements for 24 h after the last i.p. injections of MS-153 (50 mg/kg, i.p.) for the short-term study, and 10 days after the last i.p. injection to study the long-term effect of MS-153. Additionally, One-Way ANOVA (SPSS) was performed to examine the day-wise effect of treatment. Immunoblot data were statistically analyzed using One-Way ANOVA, followed by Newman-Keuls's test, for comparison among the naïve ethanol vehicle, ethanol vehicle, and ethanol MS-153 group. All statistical analyses were based on *p* < 0.05 level of significance.

## Results

### Detection of MS-153 in plasma and CSF

**Figure 2:**
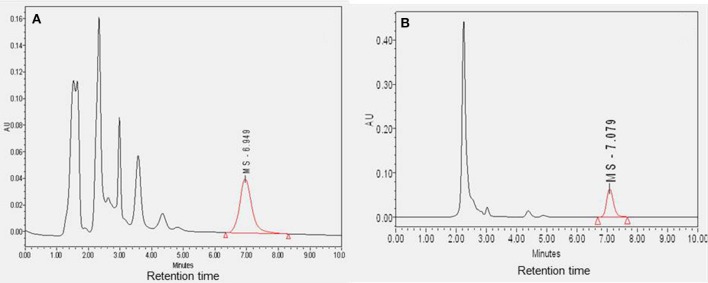
**Diagram showing the sample chromatograms of MS-153 in plasma (A) and CSF (B) (*n* = 4)**. The X-axis represents the retention time of MS-153 in minutes and the Y-axis represents the absorbance of MS-153 in absorbance units.

The plasma and CSF samples (*n* = 4) were cleaned to separate MS-153 and then analyzed using HPLC. The mean concentrations of MS-153 in plasma and CSF were found to be 14.74 ± 2.95 μg/ml and 13.52 ± 4.0 μg/ml, respectively. Sample chromatograms indicated the detection of MS-153 in plasma and CSF (Figures 2A,B). As observed in the chromatograms, the retention time of MS-153 is approximately 7 min in both plasma and CSF samples. This indicates that the HPLC method of MS-153 is very specific and no interference was observed in the extracted samples of plasma and CSF. The penetration of MS-153 into the CSF after 1 h was substantial and comparable to concomitant plasma levels. It is noteworthy that a complete *in vivo* pharmacokinetic study should be carried out in the future to determine the CSF-to-serum ratio of the areas under the curves.

### Effect of MS-153 on short-term ethanol drinking behavior, water intake, and body weight

**Figure 3:**
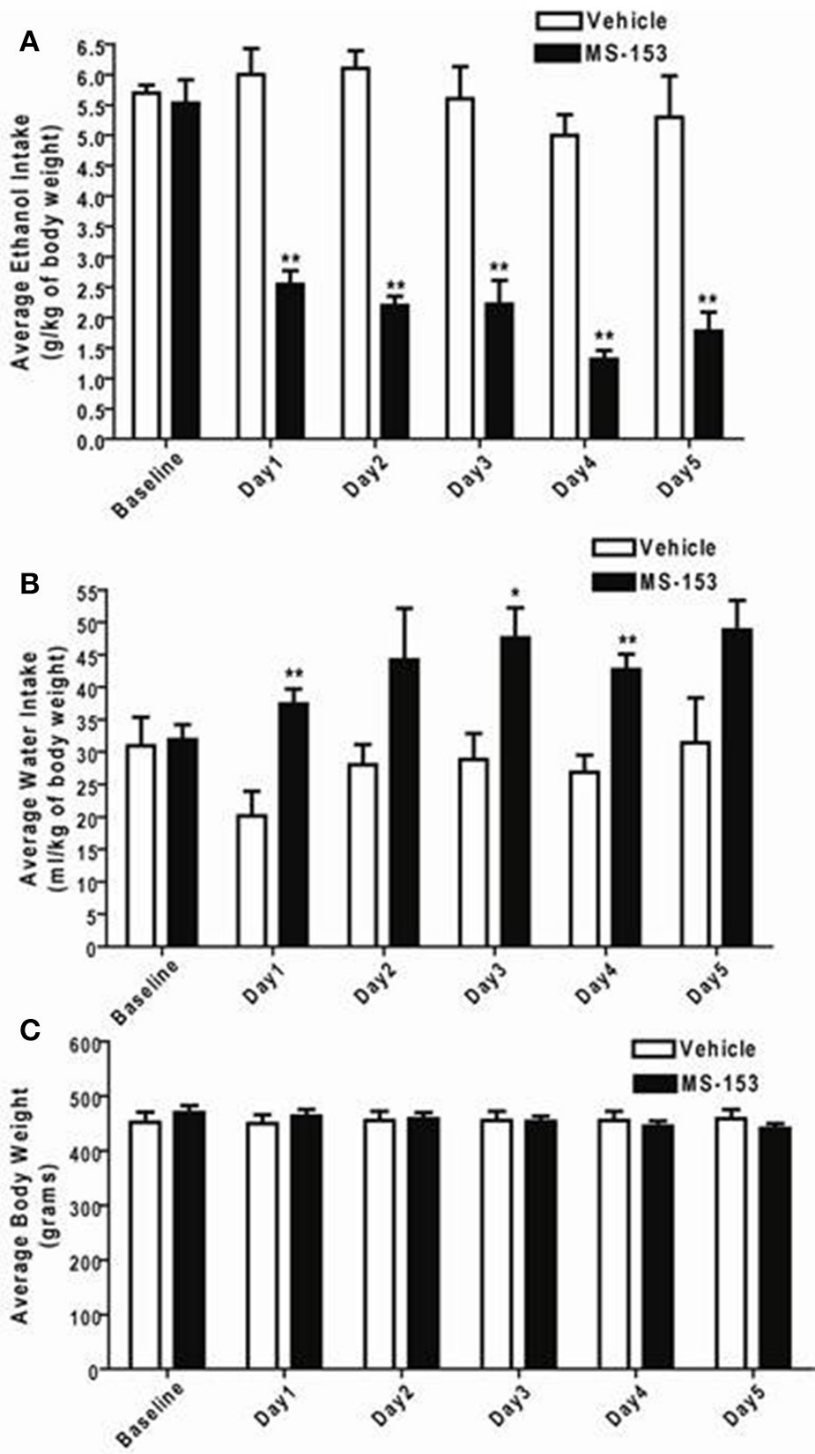
**(A)** Effects of MS-153 treatment on ethanol intake in male P rats exposed to 5 weeks of free choice of ethanol and water. Vehicle (*n* = 7), and MS-153 50 mg/kg (*n* = 5). Statistical analyses exhibited a significant decrease in ethanol consumption with MS-153 (50 mg/kg, i.p.) from Day 1 (24 h after the first i.p. injection) through Day 5 compared to the ethanol vehicle group. **(B)** Effects of MS-153 treatment on water intake in P rats exposed to 5 weeks of free choice of ethanol and water. Statistical analyses exhibited a significant increase in water consumption with MS-153 (50 mg/kg, i.p.) on Days 1, 3, and 4 compared to the ethanol vehicle group. **(C)** Effects of MS-153 treatment on body weight. Statistical analyses did not demonstrate any significant effect on body weight. Values shown as means ± s.e.m. (^*^p < 0.05; ^**^p < 0.01).

GLM repeated measures revealed a significant main effect of Day on ethanol intake [*F*_(1, 5)_ = 10.432, *p* < 0.0001] and a significant Treatment × Day interaction [*F*_(1, 5)_ = 7.476, *p* < 0.0001]. One-Way ANOVA demonstrated a significant reduction in ethanol intake among all animals treated with MS-153 at the dose of 50 mg/kg (*p* < 0.01) compared to ethanol vehicle animals (Figure 3A). In addition, GLM repeated measures revealed a significant main effect of Day on water intake [*F*_(1, 5)_ = 3.802, *p* < 0.01] but did not show a significant Treatment × Day interaction [*F*_(1, 5)_ = 2.379, *p* > 0.05]. However, One-Way ANOVA demonstrated a significant increase in water intake in the MS-153 50 group on Days 1 and 3 (*p* < 0.05) and on Day 4 (*p* < 0.01) (Figure 3B). Furthermore, GLM repeated measures revealed a significant main effect of Day on body weight [*F*_(1,5)_ = 20.024, *p* < 0.0001] and a significant Treatment × Day interaction [*F*_(1,5)_ = 49.887, *p* < 0.0001]. One-Way ANOVA did not reveal a significant effect on body weight among the ethanol vehicle and ethanol MS-153 50 groups (Figure 3C).

### Effect of MS-153 on long-term ethanol drinking behavior, water intake, and body weight

**Figure 4:**
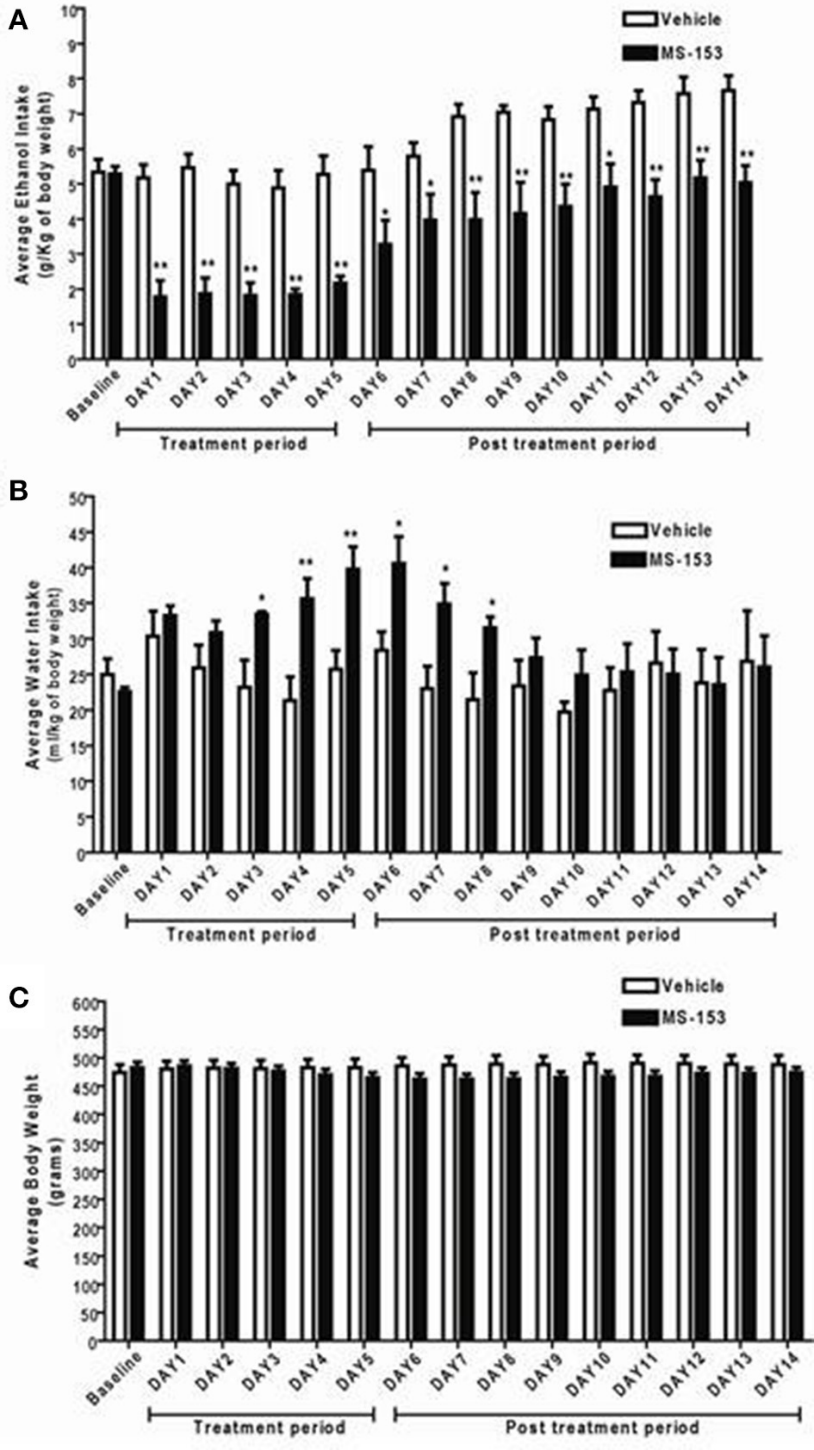
**(A)** Effects of MS-153 treatment and post-treatment on daily ethanol intake in male P rats exposed to 5 weeks of free choice of ethanol and water. Vehicle (*n* = 8), and MS-153 50 mg/kg (*n* = 8). Statistical analyses demonstrated a significant decrease in ethanol intake with the MS-153 (50 mg/kg, i.p.) treated group from Day 1 (24 h after the first i.p. injection) through Day 14 (10 days after the last injection) as compared to ethanol vehicle group. **(B)** Effects of MS-153 treatment on water intake in P rats exposed to 5 weeks of free choice of ethanol and water. Statistical analyses demonstrated a significant increase in water consumption on days 3–8. **(C)** Effects of MS-153 treatment on body weight. Statistical analyses did not demonstrate any significant effects of MS-153 on body weight. Values shown as means ± s.e.m. (^*^p < 0.05; ^**^p < 0.01).

We next examined the long-lasting effect of MS-153 on ethanol drinking behavior at the dose 50 mg/kg. GLM repeated measures revealed a significant main effect of Day [*F*_(1, 14)_ = 19.839; *p* < 0.0001] and a significant Treatment × Day interaction [*F*_(1, 14)_ = 2.814; *p* < 0.01]. One-Way ANOVA revealed a significant reduction in ethanol intake in the MS-153 50 group compared to the ethanol vehicle group from Days 1 to 14 (*p* < 0.01) (Figure 4A). Furthermore, GLM repeated measures demonstrated a significant main effect of Day on water intake [*F*_(1, 14)_ = 3.512, *p* < 0.0001] and a significant Treatment × Day interaction [*F*_(1, 14)_ = 2.39, *p* < 0.01]. One-Way ANOVA demonstrated a significant increase in water intake in the MS-153 50 group compared to the ethanol vehicle group on Days 3–8 (*p* < 0.05, *p* < 0.05) (Figure 4B). In addition, GLM repeated measures demonstrated a significant main effect of Day on body weight [*F*_(1, 14)_ = 5.147, *p* < 0.0001] and a significant Treatment × Day interaction [*F*_(1, 14)_ = 19.618, *p* < 0.0001]. However, One-Way ANOVA did not reveal a significant difference in body weight between the ethanol vehicle and ethanol MS-153 50 groups (Figure 4C).

### Effect of MS-153 on sucrose drinking behavior

**Figure 5:**
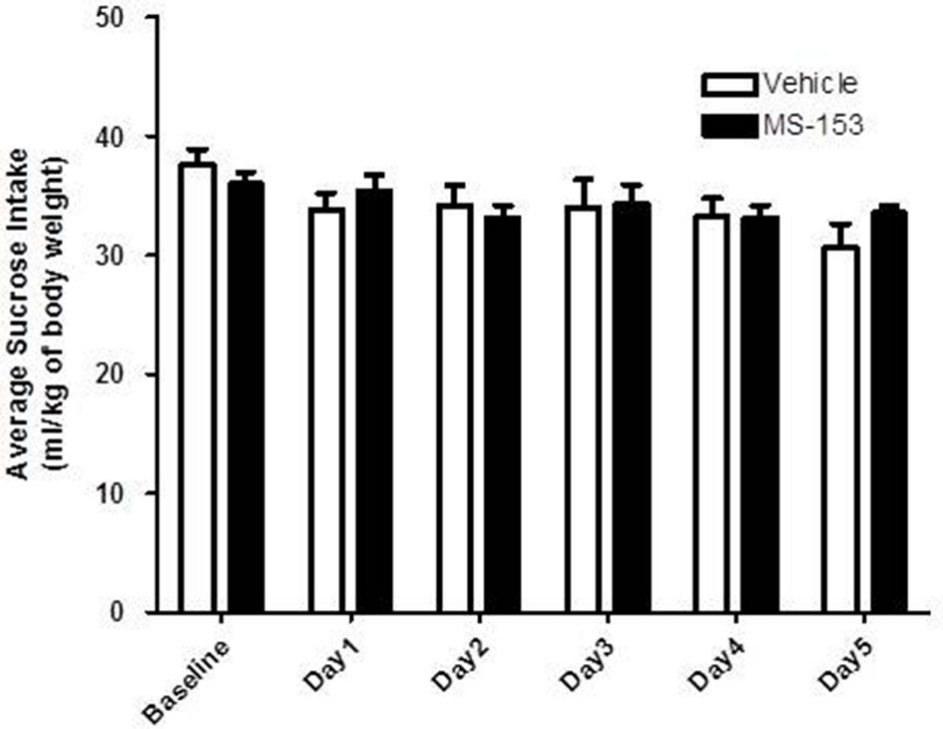
**Effects of MS-153 on sucrose intake in P rats**. Vehicle (*n* = 7) and MS-153 50 mg/kg (*n* = 5). MS-153 at the higher dose (50 mg/kg, i.p.) did not induce any effect on sucrose intake. Values shown as means ± s.e.m.

We tested the effects of MS-153 at 50 mg/kg on sucrose consumption (Figure 5). GLM repeated measures revealed a significant main effect of Day [*F*_(1, 5)_ = 7.475, *p* < 0.0001], which indicated that sucrose intake decreased in both groups across days, while, the Treatment × Day interaction was not significant [*F*_(1, 5)_ = 2.109, *p* > 0.05]. However, One-Way ANOVA did not reveal a significant difference in sucrose intake throughout the treatment period (*p* > 0.05). These data demonstrate that MS-153 did not affect sucrose intake.

### Effect of MS-153 on GLT1 expression in the NAc and PFC

**Figure 6:**
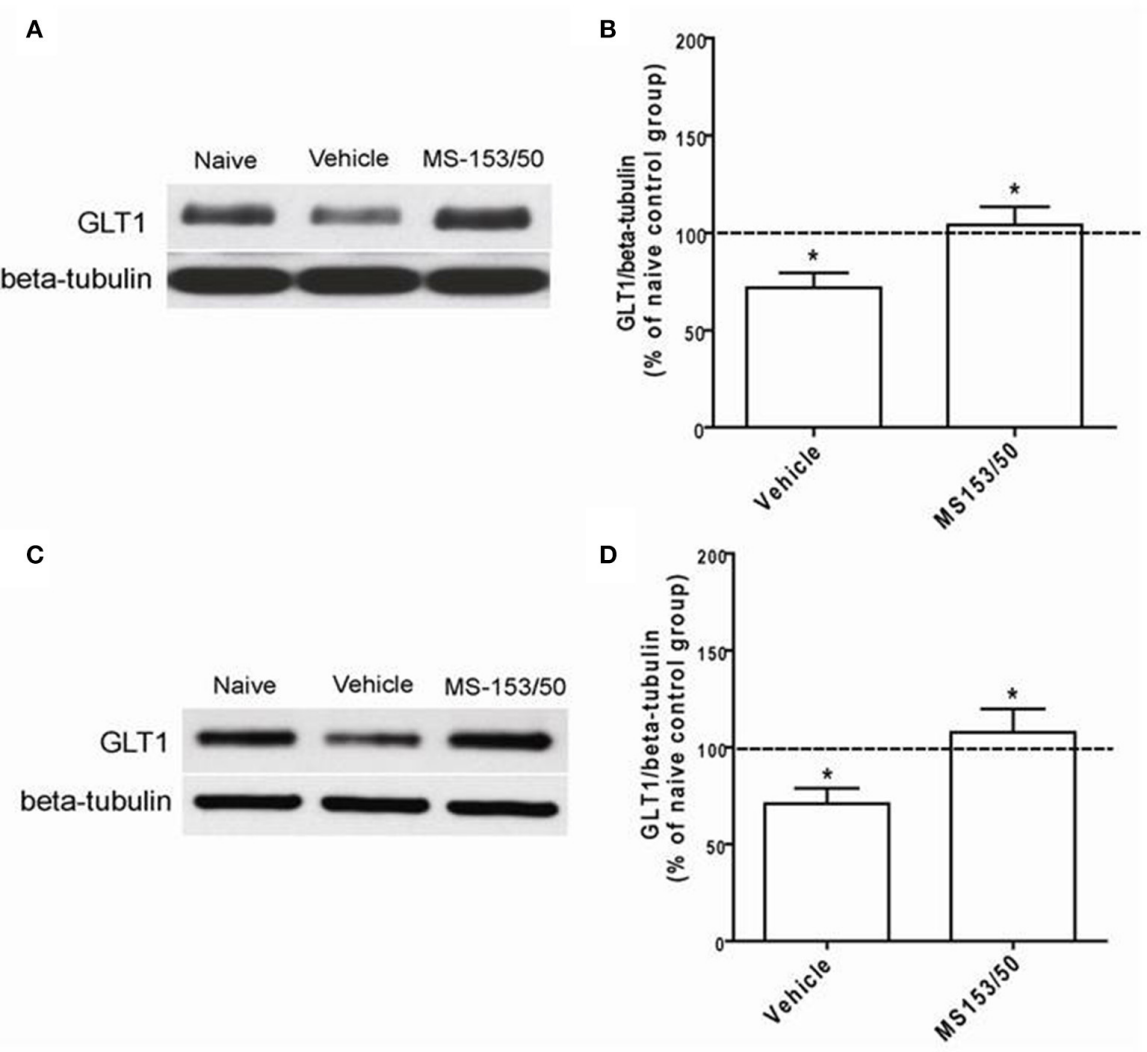
**Effects of MS-153 (50 mg/kg) on GLT1 expression level in NAc. (A,C)** Immunoblots for GLT1 and β-tubulin, which was used as a control loading protein, in the NAc after 1 day and 10 days of the last i.p. injection of MS-153, respectively. **(B,D)** Quantitative analysis revealed a significant increase in the ratio of GLT1/β-tubulin in MS-153-treated groups compared to the ethanol vehicle groups after 1 day and 10 days of the last i.p. injection of MS-153, respectively. Significant downregulation of GLT1 expression was revealed in the ethanol vehicle group compared to the naïve ethanol group. Values shown as means ± s.e.m. (^*^p < 0.05) (*n* = 5 for each group).

We further investigated the effects of MS-153 on GLT1 expression in the NAc 24 h after the last i.p. injections of MS-153 50 mg/kg (Figures 6A,B) and 10 days post-treatment (Figures 6C,D). One-Way ANOVA analyses revealed a significant main effect among the naïve ethanol vehicle, ethanol vehicle and ethanol MS-153 treated groups in the NAc [*F*_(2, 12)_ = 6.45, *p* = 0.012 for short term; and *F*_(2, 12)_ = 5.41, *p* = 0.02 for post-treatment]. The Newman-Keuls *post-hoc* test, a multiple comparison test, demonstrated a significant increase in GLT1 expression in the ethanol MS-153-treated group compared to the ethanol vehicle-treated group 24 h after the last injection of MS-153 (*p* < 0.05; Figures 6A,B) and 10 days post-treatment (*p* < 0.05; Figures 6C,D). Alternatively, statistical analyses revealed significant downregulation of GLT1 expression in the ethanol vehicle group compared to the naïve ethanol group in the NAc for both paradigms (*p* < 0.05; Figures 6B,D).

**Figure 7:**
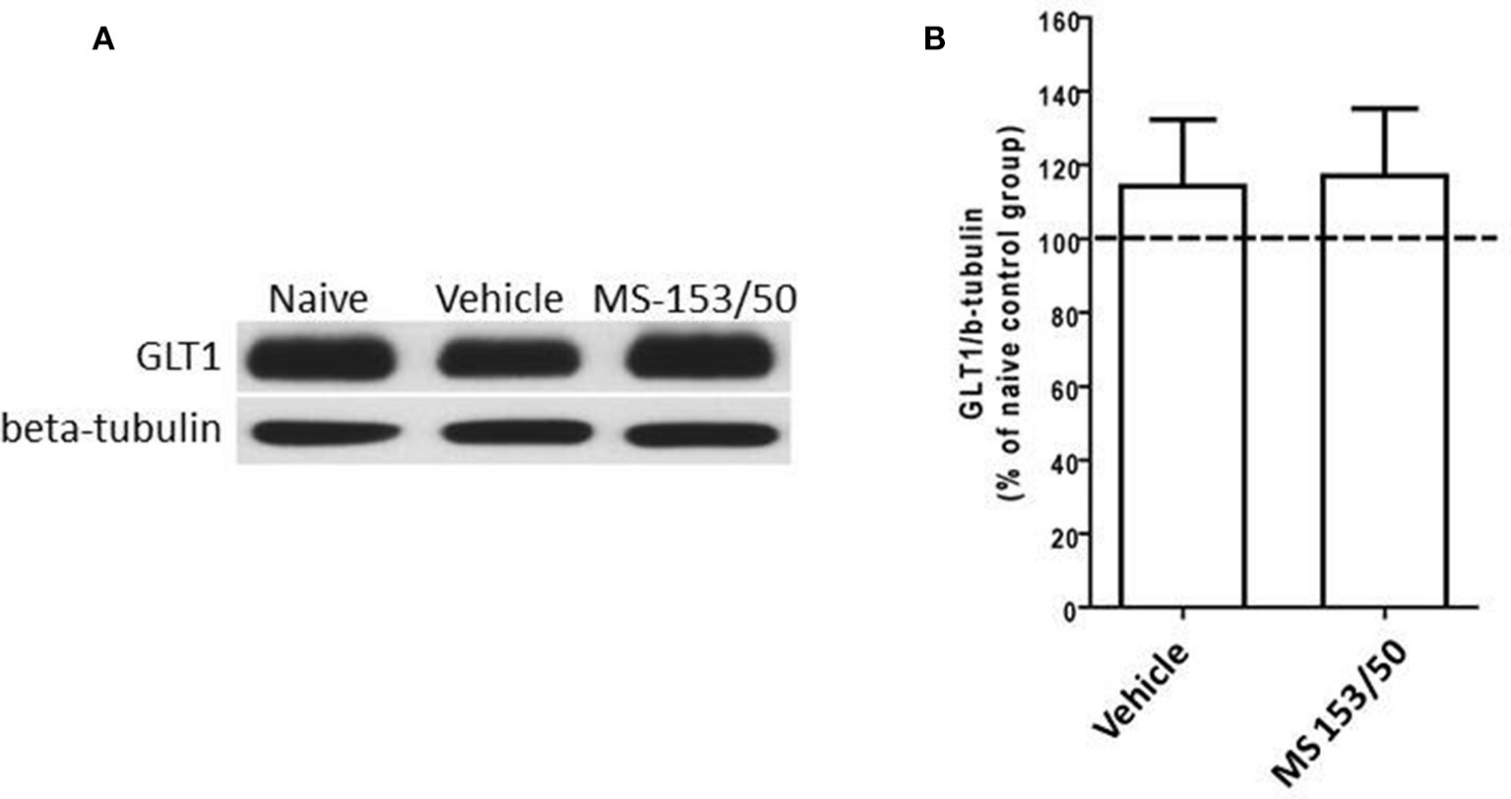
**Effects of MS-153 (50 mg/kg) on GLT1 expression in PFC. (A)** Immunoblots for GLT1 and β-tubulin, which was used as a control loading protein, in the PFC after 1 day of the last i.p. injection of MS-153. **(B)** Quantitative analysis did not reveal any significant differences in the ratio of GLT1/β-tubulin among all groups. Values shown as means ± s.e.m. (*n* = 5 for each group).

Interestingly, One-Way ANOVA analyses did not reveal any significant main effect among all groups in the PFC (Figures 7A,B). Furthermore, in accordance with a recent study from our lab (Sari and Sreemantula, 2012), we did not observe downregulation of GLT1 expression in the PFC.

### Effect of MS-153 on NF-kB-p65 and IkBα levels in the NAc and PFC

**Figure 8:**
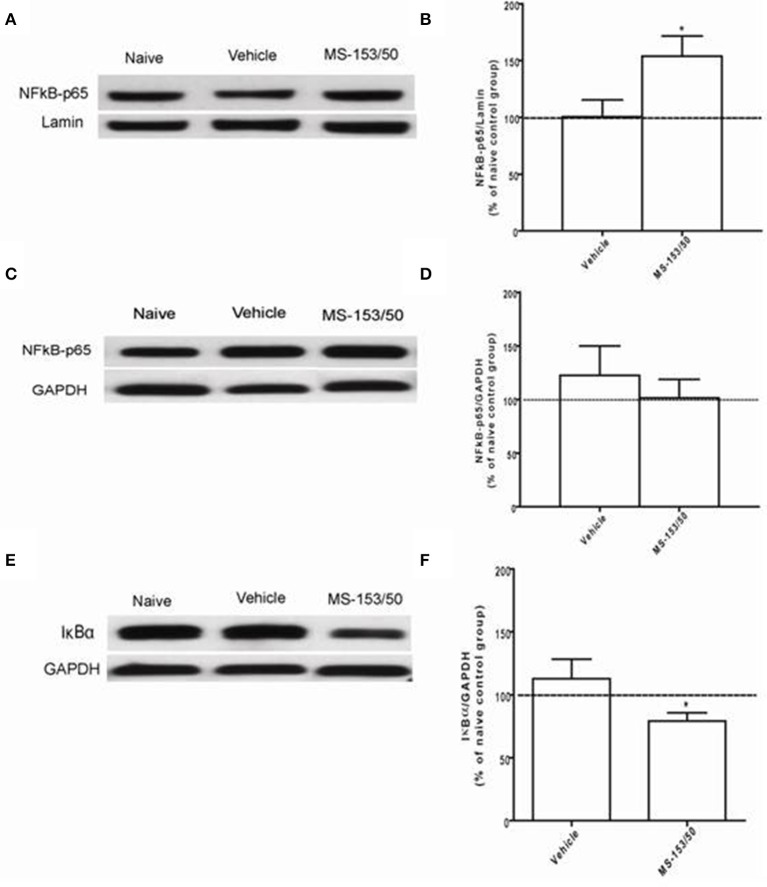
**Effects of MS-153 at 50 mg/kg (MS-153/50, *n* = 5), vehicle (ethanol vehicle group, *n* = 5), naïve (naïve ethanol vehicle group, *n* = 5) groups on NFκ B-p65 level in the NAc. (A,C,E)** Immunoblots for GAPDH and Lamin [which were used as control loading proteins for cytoplasmic extract (CE) and nuclear extract (NE) proteins, respectively], NFκ B-p65, and Iκ Bα in CE and NE. **(B,D)** Quantitative analysis of NFκ B-p65 levels in the CE and NE revealed a significant increase in the ratio of NFκ B-p65/Lamin level in the NE but not in the CE for the ethanol MS-153-treated group compared to the naïve ethanol vehicle and ethanol vehicle groups. **(F)** Quantitative analysis of Iκ Bα level in the CE revealed a significant decrease in the ratio of Iκ Bα/GAPDH level in the ethanol MS-153-treated group compared to the naïve ethanol vehicle and ethanol vehicle groups. Values shown as means ± s.e.m. (^*^p < 0.05).

We next investigated the mechanism of action of MS-153 in GLT1 upregulation. We focused on the effects of this drug on NF-kB-p65 and IkBα levels in the NAc. In addition, we determined the levels of NF-kB-p65 and IkBα as well. One-Way ANOVA analyses demonstrated a significant main effect on NF-kB-p65 levels among all groups in the nuclear fraction of the NAc [*F*_(2, 12)_ = 5.309, *p* = 0.0223]. The Newman-Keuls *post-hoc* test, a multiple comparison test, demonstrated a significant increase in the NF-kB-p65 level of the MS-153 treated group (*p* < 0.05) compared to the naïve ethanol vehicle and ethanol vehicle groups (Figures 8A,B). Statistical analyses did not show any main effect among all groups in the NF-kB-p65 level in the cytoplasmic fraction [*F*_(2, 12)_ = 0.458, *p* = 0.642] (Figures 8C,D). Furthermore, statistical analyses revealed a significant main effect on IkBα levels among tested groups [*F*_(2, 12)_ = 5.404, *p* = 0.021]. The *post-hoc* test revealed a significant decrease in the IkBα level (*p* < 0.05) of the MS-153 treated group compared to the naïve ethanol vehicle and ethanol vehicle groups in the cytoplasmic fraction of the NAc (Figures 8E,F).

**Figure 9:**
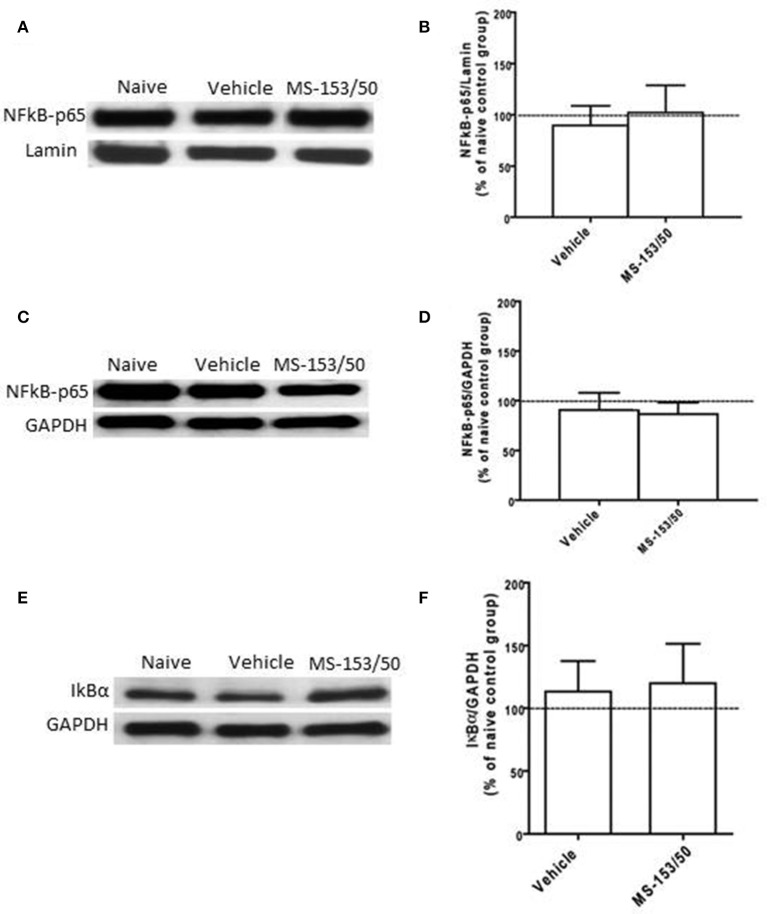
**Effects of MS-153 at 50 mg/kg (MS-153/50, *n* = 5), vehicle (ethanol vehicle group, *n* = 5), naïve (naïve ethanol vehicle group, *n* = 5) groups on NF-κ B-p65 level in the PFC. (A,C,E)** Immunoblots for GAPDH and Lamin (which were used as control loading proteins for cytoplasmic extract (CE) and nuclear extract (NE) proteins, respectively), NF-κ B-p65, and Iκ Bα in CE and NE extracts. **(B,D)** Quantitative analysis of NF-κ B-p65 levels in CE and NE did not reveal any significant change in all tested groups in the CE or NE. **(F)** Quantitative analysis of Iκ Bα level in CE did not reveal any significant change in Iκ Bα level in tested groups. Values shown as means ± s.e.m.

We further determined the levels of NF-kB-p65 and IkBα in the PFC. One-Way ANOVA analyses did not show any significant main effect among all groups in NF-kB-p65 level neither in the nuclear fraction (Figures 9A,B) nor in cytoplasmic fraction [*F*_(3, 19)_ = 0.21, *p* = 0.887] (Figures 9C,D). In addition, we did not find any significant main effect among all groups on the IkBα level in the cytoplasmic fraction (Figures 9E,F).

### Effect of MS-153 on Akt level in the NAc

**Figure 10:**
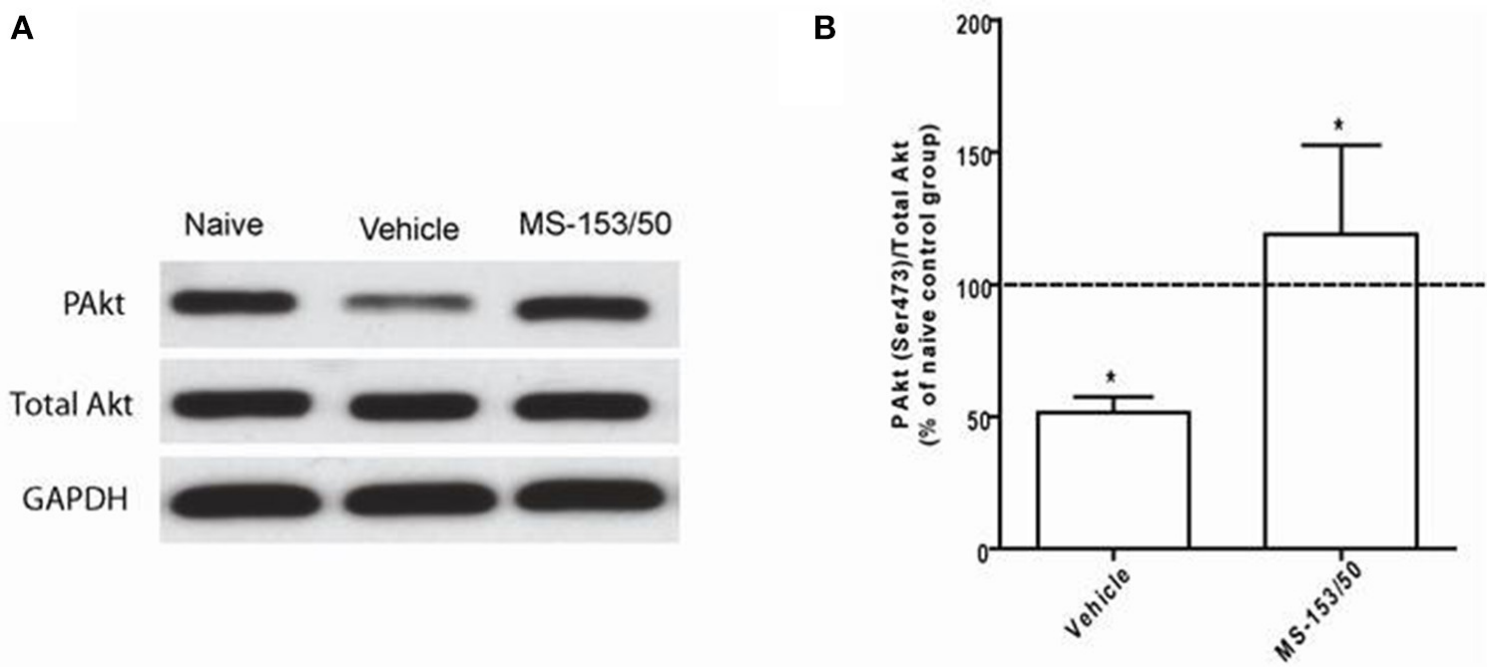
**Effects of MS-153 at 50 mg/kg (MS-153, *n* = 5), vehicle (ethanol vehicle group, *n* = 5), naïve (naïve ethanol vehicle group, *n* = 5) groups on phospho-Akt levels in the NAc. (A)** Each panel presents immunoblots for Total Akt and GAPDH, which was used as a control loading protein, and phospho-Akt (PAkt) in the NAc. **(B)** Quantitative analysis revealed a significant increase in the ratio of PAkt/total Akt in both MS-153-treated groups compared to the ethanol vehicle group. Significant downregulation of PAkt/total Akt expression was revealed in the ethanol vehicle group compared to the naïve ethanol vehicle group. Values shown as means ± s.e.m. (^*^p < 0.01).

We further explored other mechanisms of action of MS-153 in the NAc. We focused on Akt, which is known to regulate GLT1 expression *in vitro* (Li et al., 2006). One-Way ANOVA analyses demonstrated a significant main effect on the phospho-Akt level among all groups in the NAc 24 h after the last injection [*F*_(2, 12)_ = 19.07, *p* = 0.0002]. The Newman-Keuls *post-hoc* multiple comparisons test revealed a significant decrease in the Akt level (*p* < 0.001) of the ethanol vehicle group compared to the naïve ethanol vehicle group in the NAc (Figures 10A,B). Importantly, we further found that treatment with MS-153 upregulated phospho-Akt levels in the NAc compared to the ethanol vehicle group (Figures 10A,B).

### Effect of MS-153 on GLAST level in the PFC and NAc

**Figure 11:**
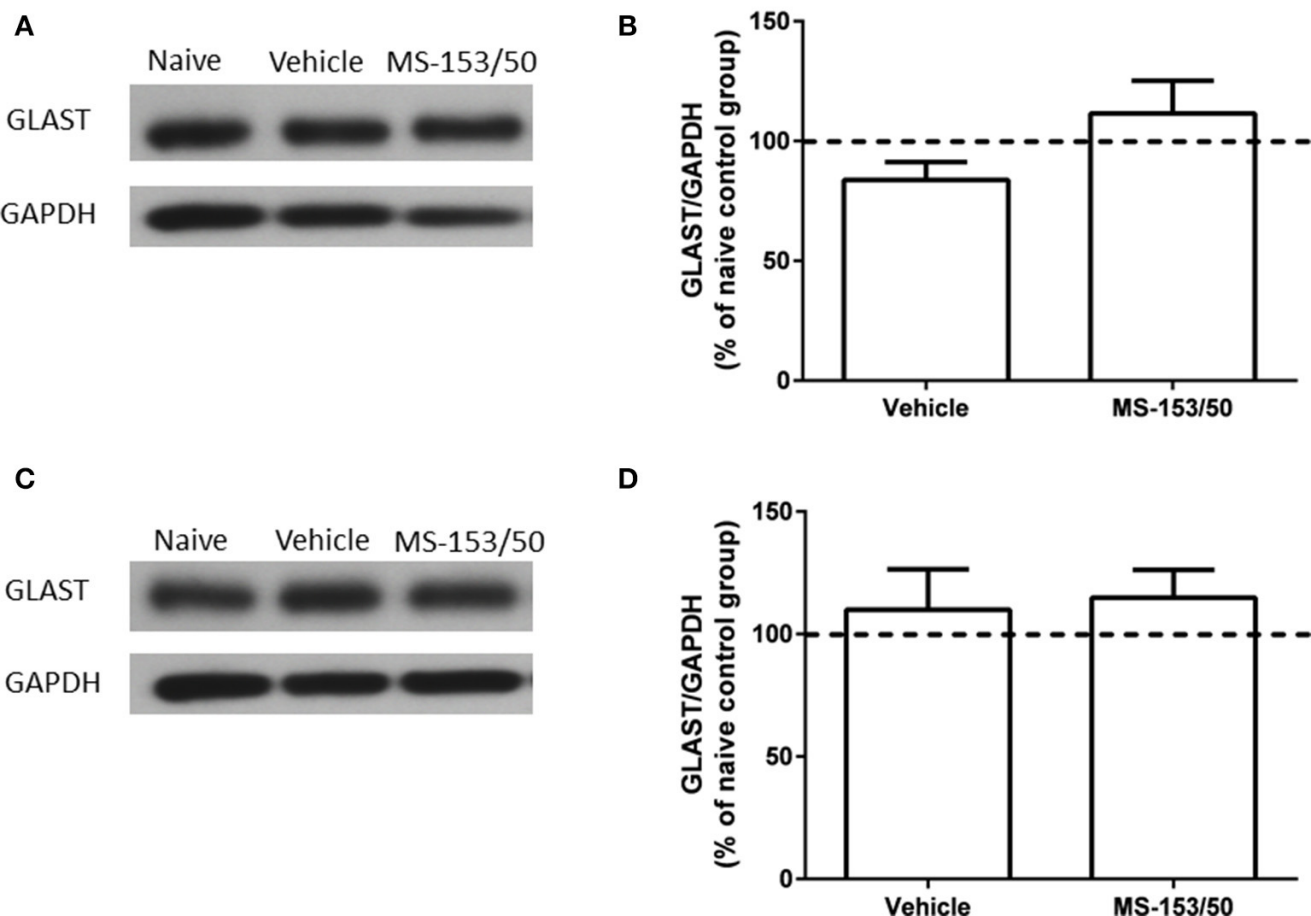
**Effects of MS-153 (50 mg/kg) on GLAST expression in PFC and NAc. (A,C)** Immunoblots for GLAST and GAPDH, which was used as a control loading protein, in the PFC and NAc, respectively. **(B,D)** Quantitative analysis did not reveal any significant differences in the ratio of GLAST/GAPDH among all groups in PFC and NAc, respectively. Values shown as means ± s.e.m. (*n* = 5 for each group).

We further tested whether MS-153 affects another astroglial transporter such as GLAST, which is co-expressed with GLT1. Thus, we examined the effects of MS-153 on GLAST level in both PFC and NAc. One-Way ANOVA analyses did not reveal any significant differences between all tested groups in PFC (Figures 11A,B) [*F*_(2, 14)_ = 0.27, *p* = 0.76], and in NAc (Figures 11C,D) [*F*_(2, 14)_ = 0.64, *p* = 0.54].

## Discussion

We report here for the first time that administration of MS-153 (50 mg/kg, i.p.) in P male rats reduced ethanol intake. We found that MS-153 reduced ethanol intake dramatically. Importantly, the effect of MS-153 on the reduction of ethanol intake was long-lasting over a period of 10 days post-treatment. MS-153 had no effect on sucrose intake, which indicates that the action of this drug is specific to ethanol intake and does not affect sucrose as an appetitive control, drinking-motivated behavior. It is noteworthy that the reduction of ethanol intake was found alongside upregulation of GLT1 in the NAc at 24 h after the last MS-153 i.p. injection and post-treatment. However, we did not find any upregulatory effects with MS-153 in PFC. We also tested GLAST, which is mainly found on the membranes of astroglial cells, where it co-expresses with GLT1 throughout the brain (Berger and Hediger, 1998). However, we did not find any significant changes in the level of GLAST in PFC and NAc neither in MS-153 treated rats nor in ethanol exposed rats, at least with the dose used in this study. This suggests the specific regulatory effect of MS-153 on GLT1 expression. Note that we did not observe any downregulation of GLAST level in both PFC and NAc in ethanol exposed rats. Accordingly, we recently reported that ceftriaxone treatment upregulated the levels of GLT1 isoforms in both PFC and NAc but no effect in GLAST level (Alhaddad et al., 2014).

We also found that MS-153 administered to P rats revealed an increase in water intake compared to vehicle-administered rats. These findings are in accordance with recent findings from our lab in the investigation of other GLT1 upregulators (Sari et al., 2011, 2013a,b; Sari and Sreemantula, 2012). We suggest that the increase in water intake is a behavioral mechanism to compensate for the decrease in ethanol intake in MS-153 treated groups.

It is noteworthy that a deficit or dysfunction of GLT1 plays a critical role in drug abuse, including alcohol and cocaine (for review see Rao and Sari, 2012). We recently reported that rats exposed to free-choice ethanol for 5 weeks showed down-regulation of GLT1 levels in the NAc but not in the PFC (Sari and Sreemantula, 2012; Sari et al., 2013b). Furthermore, i.p. injections of ceftriaxone reduced ethanol consumption in both male and female P rats (Sari et al., 2011, 2013a,b). We have further shown that ceftriaxone can also attenuate relapse-like to ethanol-drinking behavior (Qrunfleh et al., 2013). In addition, we have recently identified GPI-1046 (3-(3-pyridyl)-1-propyl (2S)-1-(3,3-dimethyl-1,2-dioxopentyl)-2-pyrrolidinecarboxylate) as another compound that reduced ethanol intake in male P rats (Sari and Sreemantula, 2012). The behavioral effects of ceftriaxone and GPI-1046 were associated with upregulation of GLT1 in the NAc. In this study, we aimed to determine the effect of a synthetic GLT1 activator, MS-153, on GLT1 level and consequently its effect in ethanol intake. The drug was shown to enhance glutamate uptake via GLT1 and prevent efflux of glutamate during cerebral ischemia (Shimada et al., 1999).

Furthermore, studies demonstrated that MS-153 inhibits the glutamatergic system, mainly by accelerating glutamate uptake; the drug had no effect on NMDA glutamate receptors, AMPA glutamate receptors, and Ca^2+^ channels (Nakagawa et al., 2005). However, it was not known whether MS-153 could upregulate GLT1 in the brain. In the present study, we further investigated whether the effect of MS-153 on the reduction of ethanol intake was associated in part with upregulation of the GLT1 level in the NAc and PFC. Surprisingly, our findings showed that MS-153 upregulated GLT1 levels in NAc but not in PFC. Note that downregulation of GLT1 level was found in NAc but not in PFC. It is clear that the drug prevented an ethanol-induced downregulation of GLT1 level in the NAc. Since there were no effects in ethanol exposure in PFC, we did not see any upregulatory action of the drug in this region. Thus, the drug has normalized the level of GLT1 affected by ethanol intake. This pharmacological action of MS-153 suggests that the drug might be considered as a modulator of the GLT1 level. Although, we demonstrated that the upregulatory effect in GLT1 has lasted 10 days after the last injection with the drug, it is noteworthy that the upregulatory effect in GLT1 level with MS-153 may not be a permanent effect.

Alternatively, similar to a recent study from our lab (Sari and Sreemantula, 2012), we also found down-regulation of GLT1 level in the NAc but not in the PFC. It is noteworthy that downregulation of GLT1 level was also found in NAc but not PFC in cocaine self-administered rats (Knackstedt et al., 2010). It is unclear in how both ethanol and cocaine have similar effect in reduction of GLT1 level. Although the neurochemistry leading to downregulation of GLT1 level is different between ethanol and cocaine, we suggest that the difference in the GLT1 levels might be due to the fact that PFC has reciprocal glutamatergic connections with other brain regions. However, NAc receives but does not send glutamatergic projections to other brain regions. It is suggested that neuroadaptations may occur due to the fact of the differences in anatomical distribution of this transporter in NAc and PFC (Danbolt, 2001; Sari and Sreemantula, 2012).

**Figure 12:**
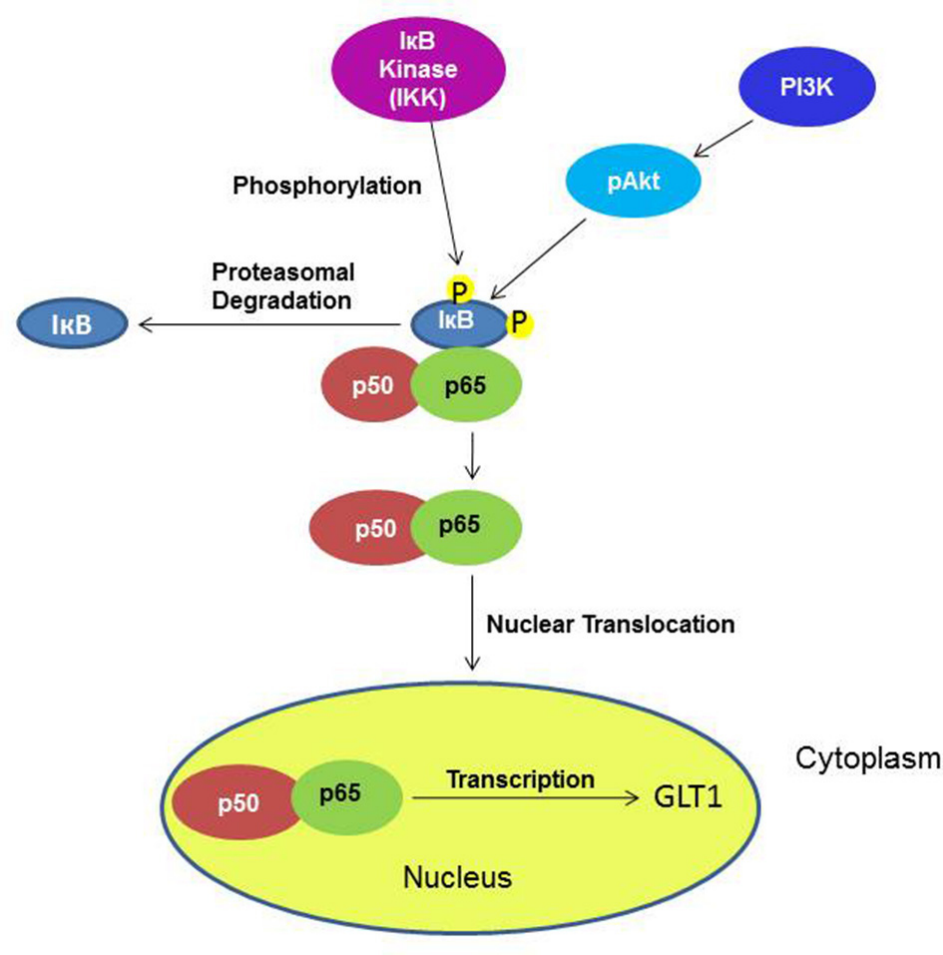
**Diagram shows the signaling pathways involving the effects of MS-153 in upregulation of GLT1**. Under an unknown mechanism, MS-153 administration may increase GLT1 transcription through phosphorylation by IkB kinase of the IkB-p50–p65 complex, which dissociated to p50–p65 and IkB. The nuclear translocation of p50–p65 can lead to increased transcription of GLT1.

Furthermore, it has been demonstrated *in vitro* that the upregulatory effect of ceftriaxone, a GLT1 upregulator, is mediated through the transcription factor NF-kB pathway, which might have direct or indirect interaction (Lee et al., 2008) (Figure 12). Additionally, it has been shown that Akt activation is found to be involved in GLT1 expression (Wu et al., 2010). Thus, we investigated whether the upregulatory effect in the NAc is mediated through these pathways. Although we did not see any upregulatory effect of GLT1 in the PFC, we investigated these pathways as well. The transcription factor, such as NF-kB, plays a critical role in various genes involved in cell growth, differentiation, and immune responses (Karin, 2006; Massa et al., 2006). The NF-kB family exists in five forms, including Rel (c-Rel), RelA (p65), RelB, NF-nB1 (p50 and its precursor p105), and NF-nB2 (p52 and its precursor p100). The transcriptional activator form is a heterodimer composed of p50 and p65, which is the most abundant activated form (Emdad et al., 2006). NF-kB is an important regulator for GLT1 expression (Sitcheran et al., 2005), which is involved in positive and negative regulation of GLT1 expression, depending on the agent administered (Su et al., 2003). Previous *in vitro* studies have shown that ceftriaxone treatment increased the GLT1 level by activating a GLT1 promoter through activation of NF-kB signaling, inducing p65 translocation by means of proteasomal degradation of IkBα (Lee et al., 2008). In this study, we demonstrated that upregulation of the GLT1 level in the NAc is associated with a significant increase in the NF-kB level of nuclear extract in MS-153-treated group. This suggests the possible action of MS-153 via the NF-kB pathway. NF-kB upregulation in the nuclear fraction was also associated with downregulation of IkBα in the cytoplasmic fraction, which indicates nuclear translocation of p65 and proteasomal degradation of IkBα. There were no changes in the NF-kB neither in nuclear fraction nor in the IkBα cytoplasmic fraction in the PFC, which correlates with no change in the GLT1 level in this region. Our results did not show change in the NF-kB level between naïve ethanol vehicle and ethanol vehicle groups in the NAc and PFC. The multi-regulatory action of NF-kB in many physiological and pathological processes may underlie these results in P rats.

We further investigated the Akt signaling pathway in the NAc since it has been shown *in vitro* that Akt is involved in GLT1 upregulation as well (Li et al., 2006; Wu et al., 2010). We found downregulation of phospho-Akt in the NAc of the ethanol exposed group compared to the naïve ethanol vehicle group. This finding is in accordance with studies that revealed downregulation of Akt after chronic treatment with cocaine (Perrine et al., 2008). Furthermore, we found that MS-153 treatment prevented the deficit in the phospho-Akt level in the NAc. It is noteworthy that the increases in the phospho-Akt level observed with MS-153 treatment correspond to the increases in GLT1 level in the NAc compared to the ethanol-exposed group.

We conclude here that MS-153 has the ability to reduce ethanol consumption in P rats, which is associated, in part, with upregulation and activation of GLT1 levels in NAc. These observations suggest that the drug may have a modulatory effect in GLT1 expression. Furthermore, we found that MS-153 increased NF-kB and phospho-Akt levels, which were well correlated with the increased GLT1 level in the NAc. Note that there might be other signaling pathways involved in MS-153-induced upregulation of GLT1 level, which are warranted investigation. It is important to note that we did not observe any effect of the drug in GLAST expression in both PFC and NAc, which indicated the specific action of MS-153 on GLT1 expression. These findings provide ample information about the potential clinical application of MS-153 as a novel drug for the treatment of alcohol dependence.

### Conflict of interest statement

The authors declare that the research was conducted in the absence of any commercial or financial relationships that could be construed as a potential conflict of interest.
